# Targeting neuroblastoma with hydroxamic acid based HDAC1 and HDAC2 inhibitors: Insights from in vitro and in vivo studies

**DOI:** 10.1007/s10637-025-01559-y

**Published:** 2025-07-10

**Authors:** Padmini Pai, Yashaswini Reddy, Ipshita Das, Babu Santhi Venkidesh, Poonam Bhandari, Pallavi Rao, Srinivas Oruganti, Keshava Prasad, Manasa Gangadhar Shetty, Kapaettu Satyamoorthy, Babitha Kampa Sundara

**Affiliations:** 1 Department of Biophysics, Manipal School of Life Sciences, Manipal Academy of Higher Education, Manipal, 576104 India; 2 Department of Radiation Biology & Toxicology, Manipal School of Life Sciences, Manipal Academy of Higher Education, Manipal, 576104 India; 3 Department of Cell and Molecular Biology, Manipal School of Life Sciences, Manipal Academy of Higher Education, Manipal, 576104 India; 4 Dr. Reddy’s Institute of Life Sciences, University of Hyderabad Campus, Gachibowli, Hyderabad 500046 India; 5 Shri Dharmasthala Manjunatheshwara (SDM) University, Manjushree Nagar, Sattur, Dharwad 580009 India

**Keywords:** Histone deacetylase, Hydroxamic acid, Isoform selective inhibitors, Neuroblastoma, Xenograft model

## Abstract

Histone deacetylases (HDACs) serve a crucial function in transcription regulation, and their dysregulation is linked to numerous diseases, including cancer. Among them, HDAC1 and HDAC2 are particularly significant in neural progenitors and are frequently overexpressed in neural-derived cancers. HDAC inhibitors (HDACis) have shown promise in overcoming chemoresistance by restoring tumor suppressor function in neuroblastoma cells. However, the lack of selectivity in existing HDACis presents challenges, highlighting the need for isoform-selective inhibitors to reduce side effects. This research investigated the anticancer properties of a newly synthesized hydroxamic acid derivative, emphasizing its selective HDAC1 and HDAC2 inhibition and strong antitumor activity. Our findings demonstrated that the newly developed hydroxamic acid analogues, **3A** and **3B**, effectively inhibited neuroblastoma cells (SH-SY5Y) proliferation, with IC_50_ values of 8.49 µM and 4.44 µM, respectively, comparable to suberoylanilide hydroxamic acid (SAHA) with IC_50_ of 0.91 µM. Additionally, compounds **3A** and **3B** exhibited potent HDAC inhibition. Compound **3A **selectively inhibited HDAC2 with an IC_50_ value of 0.89 μM, while compound **3B** showed dual inhibition of HDAC1 and HDAC2, with IC_50_ values of 0.44 μM and 1.94 μM, respectively. Compound **3B** triggered cell cycle arrest in the G2/M phase, reduced colony formation efficiency, and altered cellular architecture upon treatment, further highlighting its anticancer potential. In an in vivo xenograft model, compound **3B** significantly decreased tumor growth and tumor weight, highlighting its potential as an effective anticancer agent for neuroblastoma, offering both isoform-selective HDAC inhibition and potent anticancer effects.

## Introduction

Neuroblastoma, a prevalent extracranial solid tumor originating from the sympathetic nervous system, accounts for approximately 15% of juvenile cancer-related deaths [1]. Despite multiple treatment options, including chemotherapy, multi-target therapy, immunotherapy, and radiotherapy, high-risk neuroblastoma cases often have poor outcomes and may develop chemoresistance [2]. The aggressiveness of neuroblastoma is frequently linked to the oncogenic driver MYCN gene, with its encoded protein N-Myc plays a pivotal role in tumor development [3]. However, there are currently no therapeutic treatments that target N-Myc activity directly. Alternative therapeutic strategies have focused on epigenetic modulators, such as histone deacetylases (HDACs), histone acetyltransferases, and methyltransferases [4]. HDACs are crucial epigenetic regulators in various cancers, including neuroblastoma.

Although there are no therapeutic treatments that target N-Myc directly, other treatment options have focused on epigenetic modulators, such as histone deacetylases (HDACs), histone acetyltransferases, and methyltransferases [4]. HDACs are important epigenetic regulators in different cancers, including neuroblastoma. In HDAC isoforms, HDAC1 and HDAC2 have been a target of research for its role in memory formation and cognitive disorders, making it a crucial target for potential treatment option for neuroblastoma [5]. However, HDAC1 and HDAC2 isoforms play important role in regulating neuroblastoma,there is currently a lack of specific inhibitors targeting these isoforms. Currently, pan-inhibitors are utilized to treat different types of cancer [6]. Research indicates that HDAC1 and HDAC2 inhibitors have the potential to effectively suppress neuroblastoma cell proliferation, induce cell cycle arrest, increase cellular differentiation, and initiate apoptosis [7, 8]. Over the years, different HDACis have been developed and characterized as pan-inhibitors, class-selective inhibitors, and isoform-selective inhibitors, based on chemical structures and selectivity profiles [9, 10]. The isoform-selective inhibitors have gained attention due to limitations of pan-inhibitors, which cause side effects such as thrombocytopenia, headache, and nausea [11]. After several years of research, five HDACis have been developed and approved for clinical use: SAHA, Romidepsin, Panobinosat, Belinostat and Givinostat [12]. The pharmacophore model for HDACis include zinc-binding group (ZBG), which chelates the zinc ion in the active site, a linker, and a cap group [13, 14]. The hydroxamic acid group is the most used and potent ZBG, it effectively binds to the HDAC active site [15]. This research focuses on developing novel hydroxamic acid-based derivatives as potent HDAC1 and HDAC2 inhibitors for neuroblastoma treatment. We aimed to study the efficacy of these inhibitors both in vitro and in vivo to understand their potency and advance the development of more efficient neuroblastoma therapies with reduced side effects and improved treatment precision.

## Materials and methodology

SAHA and CI-994 are used as positive controls and purchased from Sigma Aldrich. Propidium iodide (PI) stain was obtained from SRL chemicals. The Annexin V-FITC apoptosis staining/detection kit was procured from Abcam. Actin phalloidin stain was purchased from Sigma Aldrich. Dulbecco’s Modified Eagle Medium (DMEM) was acquired from Himedia. HDAC1 (Catalogue No. 50051) and HDAC2 (Catalogue No. 50002) were purchased from BPS Biosciences.

### Chemical structure

**Fig. 1:**
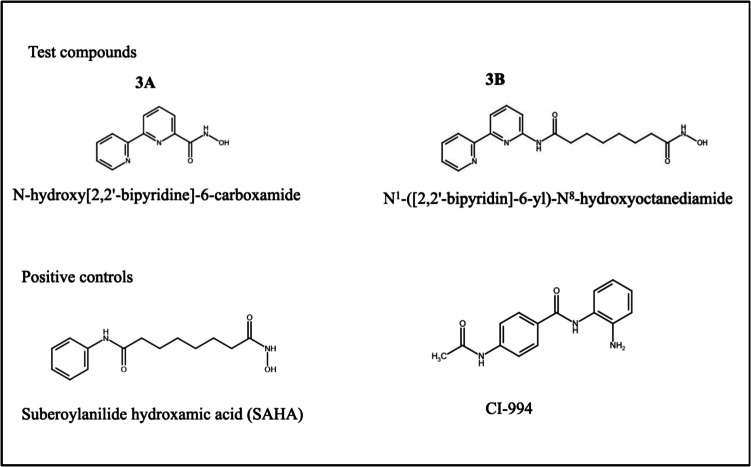
Chemical structures of the test compounds (**3A** and **3B)** and positive controls (SAHA and CI-994)

Two novel hydroxamic acid derivatives (**3A**—*N*-hydroxy[2,2'-bipyridine]-6-carboxamide and **3B**—*N*^1^-([2,2'-bipyridin]-6-yl)-*N*^8^-hydroxyoctanediamide), which are analogues of SAHA, were designed, synthesized, and characterized previously in our lab. The compounds are patented by Manipal Academy of Higher Education, Manipal (Patent ID No. 202441019540). Preliminary data support that these compounds are potent HDACis and docking study shows these compounds as potent HDAC1 and 2 inhibitors. The structures of the compounds are given below along with the positive controls (SAHA and CI-994) **(**Fig. 1**)**.

### Biological evaluation

SH-SY5Y, human neuroblastoma cell line was obtained from American Type Culture Collection (ATCC) and was cultured in DMEM/F12 low glucose media supplemented with 10% FBS. The cell line was incubated at 37 °C with 5% CO₂ in an Eppendorf CellXpert® C170, and the culture media was replaced every 48–72 h to support ideal growth. BALB/c male nude mice was procured from ACTREC Mumbai.

#### Cytotoxicity assay

SH-SY5Y cells were loaded at 100,000 cells/mL, in a 96-well plate and was incubated for 24 h. Different concentrations of drugs were dissolved in dimethyl sulfoxide (DMSO), and the cells were treated with different concentrations of test compounds and maintained for 48 h. After that, 5 mg/mL of MTT solution was added and incubated for 4 h. The MTT added media was discarded, and DMSO was added in each well. Absorbance was recorded at 570 nm and 630 nm using a TECAN multiplate reader (Austria).

#### Cell cycle analysis

SH-SY5Y cells were cultured and plated 1,000,000 cells/mL in a 10-cm plate. After reaching 60% confluency, they were exposed with different concentration of the test compounds and maintained for 48 h. After that, cells were washed with PBS, trypsinized, and fixed with 70% ethanol and incubated for 1 h. Followed by, centrifugation at 106 rcf for 10 min at 4 °C. RNase was added to pellet and kept for 3 h in water bath at 37 °C, then stained with PI and incubated for 30 min. The readings were recorded via Beckman Coulter CytoFLEX flow cytometer (California, USA).

#### Apoptosis assay

Apoptosis was assessed following drug treatment using flow cytometry. 30,000 cells were plated per 3 mL of media in a 6-cm plate, maintained for 24 h. Cells were treated with various concentration of the test compounds and incubated for 48 h. Then, cells were collected, centrifuged, and to the supernatant binding buffer was added and stained using PI and Annexin V. The cells were incubated for 10–15 min in dark, and apoptosis analysis was performed using the Partec CyFlow Space flow cytometer with FloMax software (Germany).

#### Colony formation assay

SH-SY5Y cells were seeded (500 cells/well) in a 6-cm plate. After 24 h, cells were exposed with IC_50_ concentrations of the test compounds, maintained for 48 h. Media was changed every 3 days. After 14 days, colonies were stained using 0.4% crystal violet for 10 min and washed twice with Milli-Q; then, colonies were counted.

#### Confocal imaging

SH-SY5Y cells were cultured on coverslip placed in a 6-cm plate and were treated with different concentration of the test compounds, which were incubated for 48 h. After that, the coverslips were rinsed with PBS to eliminate residual media, and then, the coverslips were further processed. First cells were fixed using 4% paraformaldehyde; blocking was performed using 5% bovine serum albumin and 0.5% Triton-X for 1 h. The cells were further stained with actin phalloidin for 45 min, followed by stain removal and two washes with PBS. The cells were subsequently stained with DAPI stain for 10 minutes, after which another PBS wash was performed. Then, the coverslips were mounted on glass slide and visualized using Leica SP8 confocal microscope via the Leica Application Suite (Germany).

#### HDAC1 and HDAC2 activity assay

In a multiwell plate (black), different concentrations of test compounds, HDAC assay buffer, and HDAC1/HDAC2 proteins were mixed and incubated for 30 min. A fluorescent substrate (Boc-Lys (Ac)-AMC) was then added to the reaction mixture and continuously shaken at room temperature for 2 h. To stop the reaction, a developer solution consisting of trypsin, Trichostatin A (TSA) (0.2 mM), and HDAC assay buffer was added and the mixture was left at room temperature for 10–15 min. Fluorescence intensity was recorded using a TECAN multiplate reader, with an excitation wavelength of 354 nm and emission wavelength of 450 nm.

#### Western blotting

SH-SY5Y cells were plated 200,000 cells/well in a 6-well plate and treated with different concentration of the test compounds and incubated for 24 h. After that cells were lysed in RIPA buffer, lysate was collected and centrifuged to collect the protein. Protein samples were separated using SDS-PAGE. Subsequently, proteins were then transferred onto a nitrocellulose membrane for 2 h. The blocking was done by 5% BSA and incubated with CST antibodies, including acetyl-histone H3 (Lys9), acetyl-histone H4 (Lys8), Bax, Bcl-2, and β-Actin at a 1:5000 dilution. After that, it is incubated with secondary anti-rabbit antibodies at a 1:5000 dilution. The protein bands were visualized via the iBright™ CL1500 Imaging System (USA).

#### Antitumor activity

Animal experiments were carried out in accordance with ethical guidelines with prior approval from the Institutional Animal Ethics Committee, Manipal Academy of Higher Education, Manipal (IAEC No. IAEC/KMC/82/2023). All experiments were carried out at the animal house facility, Manipal Academy of Higher Education, Manipal. For this study, male BALB/c male nude mice aged 6–8 weeks with body weights of approximately 25–30 g were used. Xenograft models were generated by subcutaneously injecting 3 × 10⁶ SH-SY5Y cells into the flank region of the mice. When the tumor volumes reached approximately around 100–300 mm^3^, the mice were randomly allocated to two groups control and treatment group (*n* = 3) for further studies. The treatment group received oral administration of compound **3B** at daily dose of 50 mg/kg for 14 days. The drugs were administered using a solution containing 10% DMSO and 10% cremophor in Milli-Q water. The tumors were excised, and weight was recorded. The excised tumors were then subjected to histopathological analysis to evaluate the in vivo antitumor efficacy of compound **3B** against neuroblastoma cells.

#### Histopathology

Hematoxylin and eosin (H&E) staining was conducted to examine tissue pathology. Paraformaldehyde (4%) was used to first fix the tissue samples, followed by dehydration and paraffin embedding. Using a microtome, the samples were sliced into 4 µm thick vertical sections. After the dewaxing process, the sections were stained using Mayer’s hematoxylin for 5 min, followed by eosin-phenoxine staining for 2 min. Subsequently, the stained sections were dehydrated and sealed using neutral resin. A coverslip was placed on each slide, and the processed slides were observed under an LX-500 LED trinocular research microscope (Labomed). Images were captured using a MiaCam CMOS AR 6pro microscope camera connected to the Image AR Pro software. Histopathological analysis was carried out to assess tumor morphology, including necrosis, tumor cell presence and mitotic figures.

## Results

### Cytotoxicity study

**Fig. 2:**
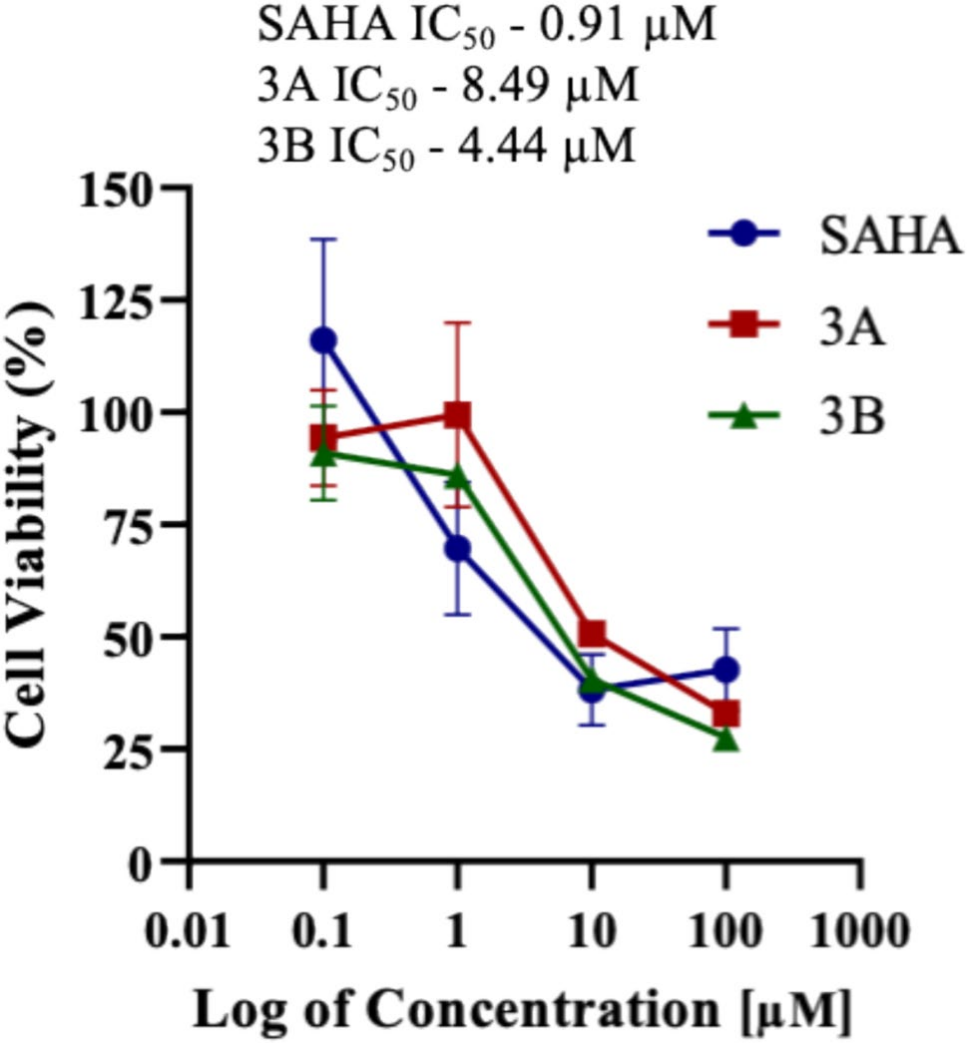
The graph shows the percentage of cell viability versus the logarithm of the concentration of compounds SAHA, **3A**, and **3B** at 48 h in SH-SY5Y cells

Cell viability was evaluated by MTT Assay of compounds **3A** and **3B**, on human neuroblastoma (SH-SY5Y). The assay determined the IC_50_ values of the test compounds following 48 h of drug exposure (Fig. 2). Compounds **3A** and **3B** had IC_50_ values of 8.49 µM and 4.44 µM, respectively, whereas positive control SAHA showed an IC_50_ value of 0.91 µM.

### Cell cycle analysis

**Fig. 3:**
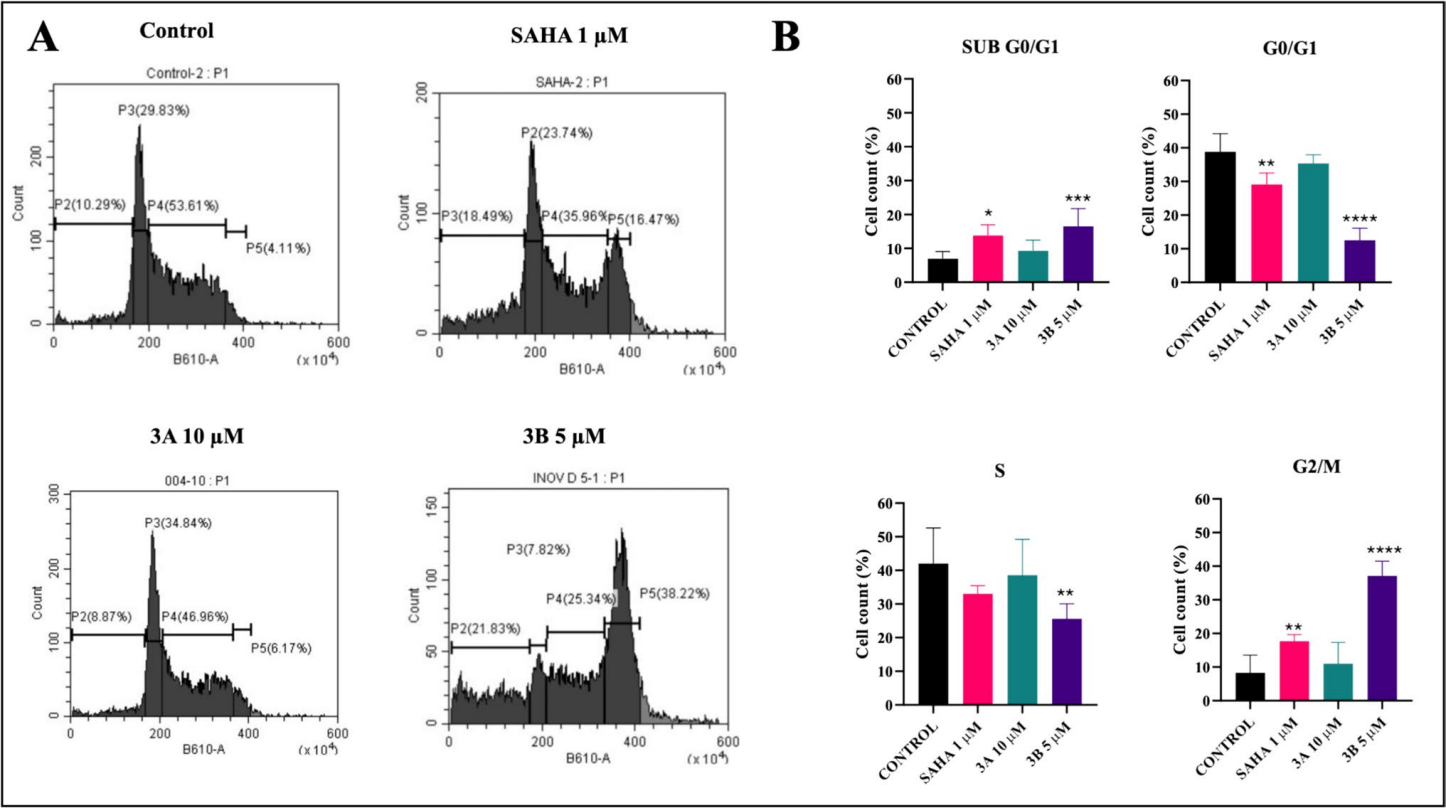
Representative flow cytometric histograms and graphs showing alterations in cell cycle as measured at 48 h after compound treatment. **A** Cell cycle distribution (P2: SUB G0/G1, P3: G0/G1, P4: S and P5: G2/M). **B** Cell cycle analysis of SH-SY5Y cells treated with the IC_50_ concentrations of SAHA, **3A**, and **3B** (1 µM, 10 µM, and 5 µM, respectively) for 48 h. *p < 0.05, **p < 0.01, ***p < 0.001, ****p < 0.0001 (*vs*. the control) determined with one-way ANOVA, Dunnett’s multiple comparison

Treatment with **3A**, and SAHA at their respective IC_50_ concentrations for 48 h resulted in an increased cell population in different phases. The results of the cell cycle analysis of 3B exhibited potent inhibitory effects by causing arrest in the G2/M phase, with 37% of the cells accumulating in this phase surpassing the outcomes observed in the untreated and positive control groups (Fig. 3).

### Apoptosis assay

**Fig. 4:**
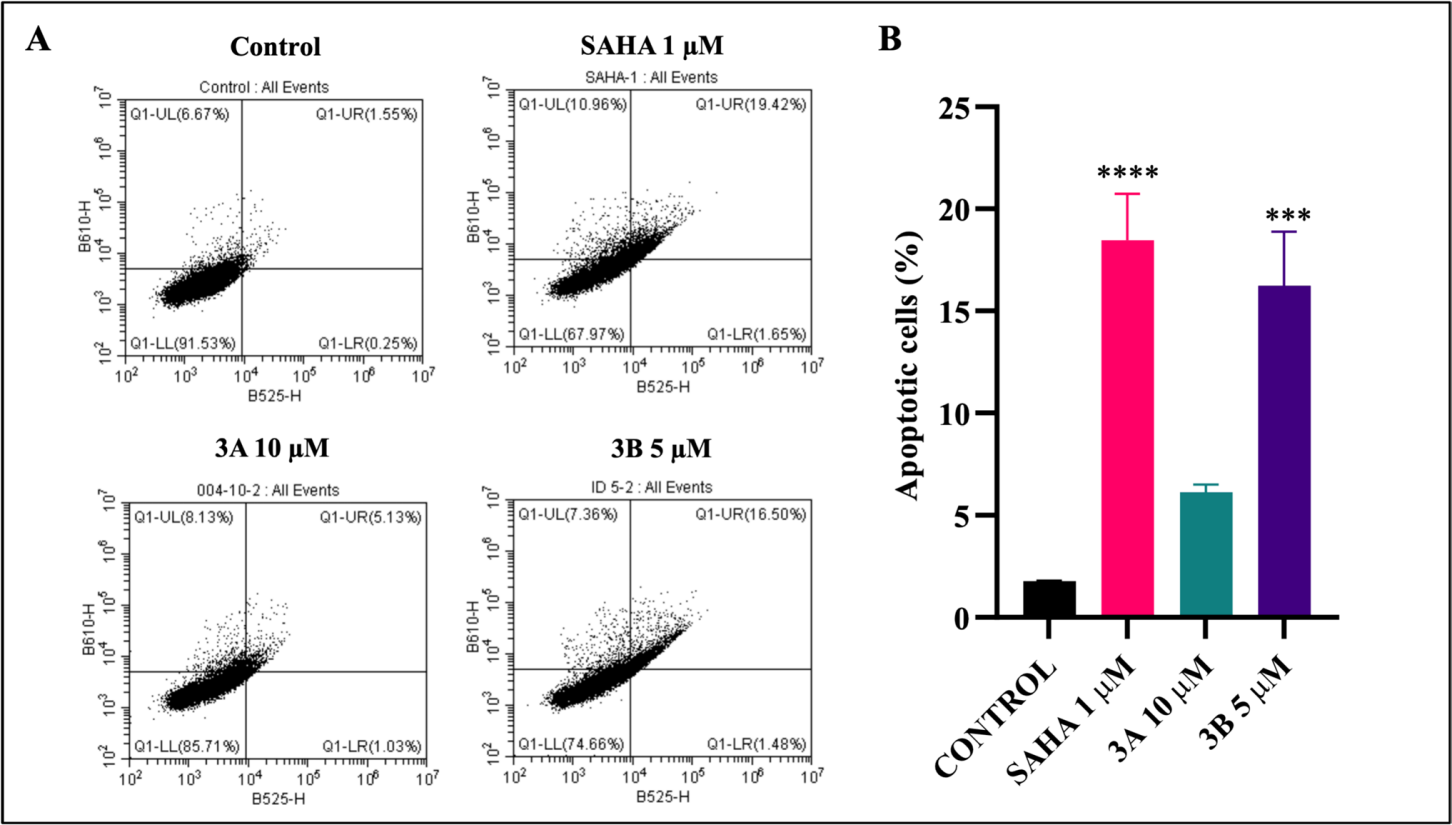
**A** Apoptotic cell distribution of SH-SY5Y cells at IC_50_ concentration of SAHA, **3A**, and **3B** (1 µM, 10 µM, and 5 µM, respectively). **B** Quantitative analysis of the treated groups compared to the control total apoptotic phases. ***p < 0.001, ****p < 0.0001 (*vs.* the control) determined with one-way ANOVA Dunnett’s multiple comparison test

After exposed with compounds **3A**, **3B**, and SAHA at IC_50_ concentration induced apoptosis in neuroblastoma cells. Notably, compound **3B** significantly raised percentage of apoptotic cells to 15% compared to the untreated group (Fig. 4). Additionally, **3B** compound demonstrated an apoptotic effect similar to the SAHA. These findings suggest that **3B** possess potent anticancer properties, with the potential to inhibit neuroblastoma cell growth effectively.

### Colony formation assay

**Fig. 5:**
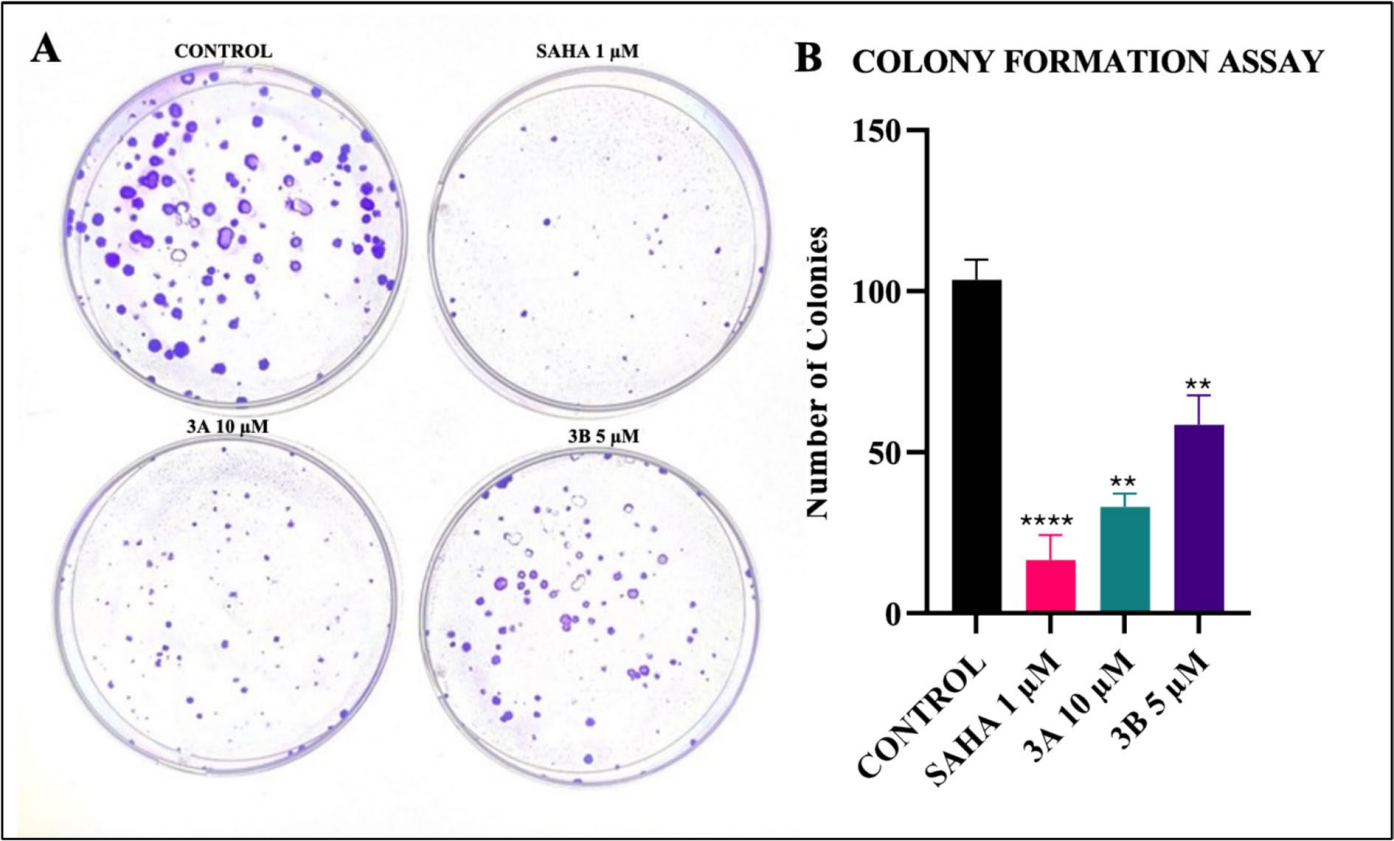
**A** SH-SY5Y cell colonies at the IC_50_ concentrations of SAHA, **3A**, and **3B** (1 µM, 10 µM, and 5 µM, respectively) for 48 h. **B** Graph showing the number of colonies on treatment with SAHA, **3A**, and **3B** for 48 h. **p < 0.01, ****p < 0.0001 (*vs*. the control) determined with one-way ANOVA, Dunnett’s multiple comparison test

Upon treating with compounds at their IC_50_ concentrations for 48 h, significant reductions in clonogenic efficiency were observed, with **3A** and **3B** exhibiting the significant inhibition, compared with SAHA (Fig. 5).

### Cell morphology

**Fig. 6:**
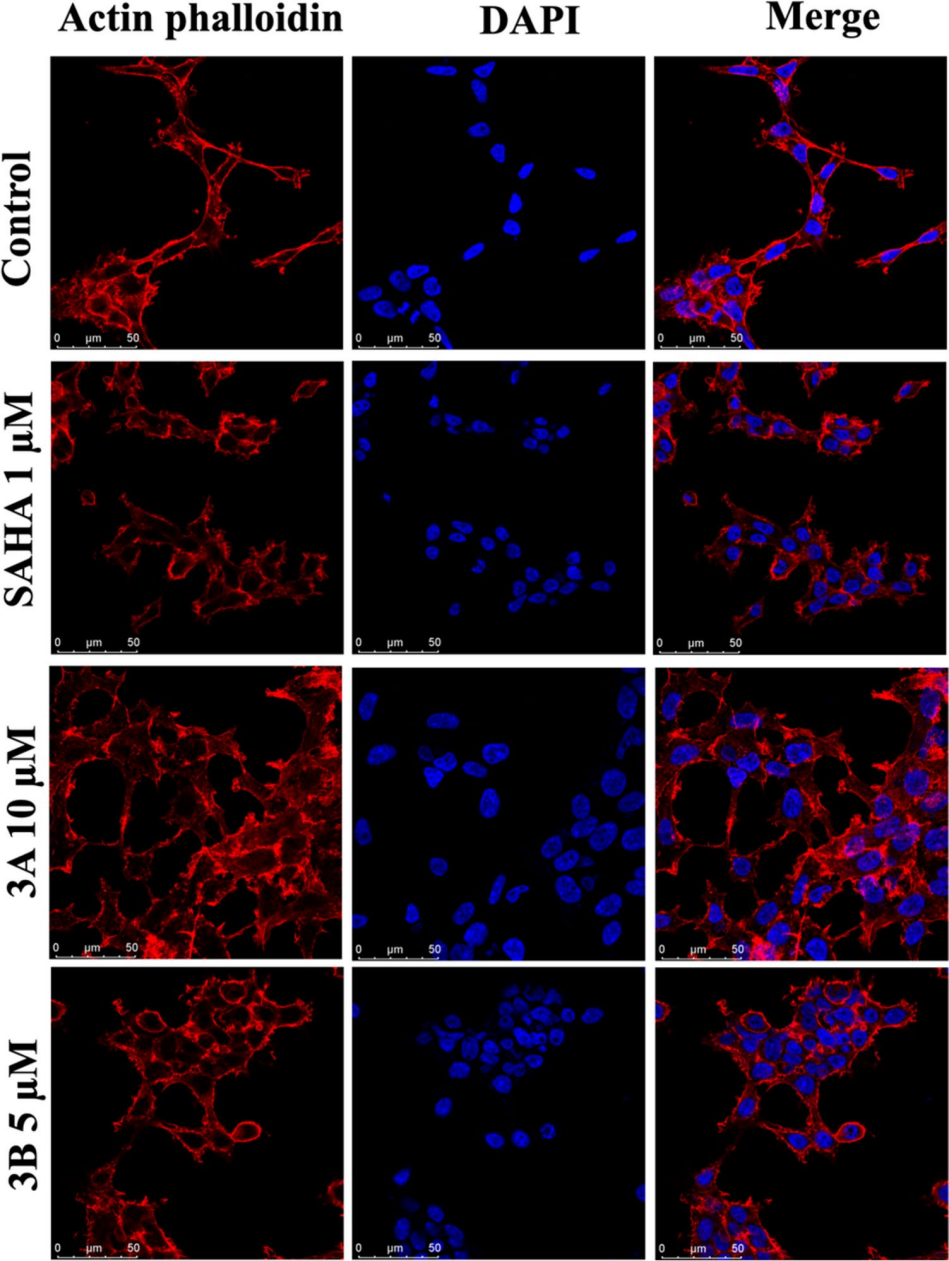
Images of nuclear and cyto-skeleton staining via confocal imaging of the SH-SY5Y cells treated with IC_50_ concentrations of SAHA, **3A**, and **3B** (1 µM, 10 µM, and 5 µM, respectively) for 48 h (scale bars: 50 μm)

Morphological characteristics were examined using confocal imaging after treating cells with IC_50_ concentrations. Two stains were used in the analysis: DAPI, a nuclear stain, and actin phalloidin, a cytoplasmic stain that highlights microtubules. Both the **3A** and **3B** compounds exhibited nuclear bulging, as shown by the DAPI staining results (Fig. 6). Actin phalloidin staining revealed changes in actin filament distribution.

### HDAC1 and HDAC2 activity assay

**Fig. 7:**
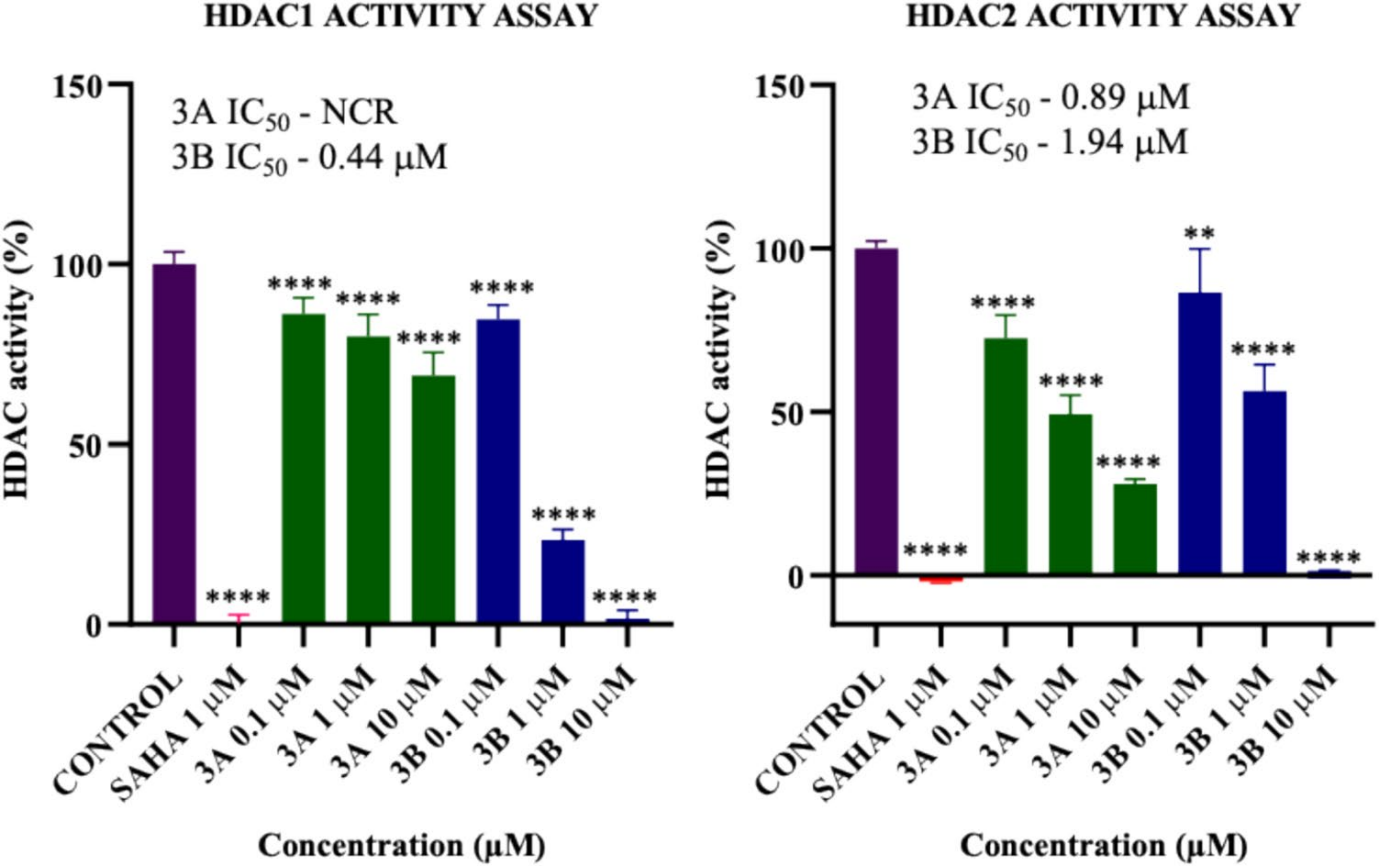
Effects of SAHA, **3A**, and **3B** compounds on HDAC1 and HDAC2 activity. **p < 0.01, ****p < 0.0001 (*vs*. the control) determined with one-way ANOVA, Dunnett’s multiple comparison test (NCR – not in concentration range)

HDAC1 and HDAC2 inhibition was assessed via a HDAC activity assay. The inhibitory effects of **3A**, **3B**, and SAHA were evaluated through a fluorescence-based assay, employing HDAC1 and HDAC2 proteins and a fluorogenic acetylated histone peptide fragment as the substrate. **3B** show HDAC1 inhibition with IC_50_ 0.44 μM respectively. **3A** and **3B** show the HDAC2 inhibition with IC_50_ values 0.89 μM and 1.94 μM, respectively (Fig. 7).

### Western blot analysis

**Fig. 8:**
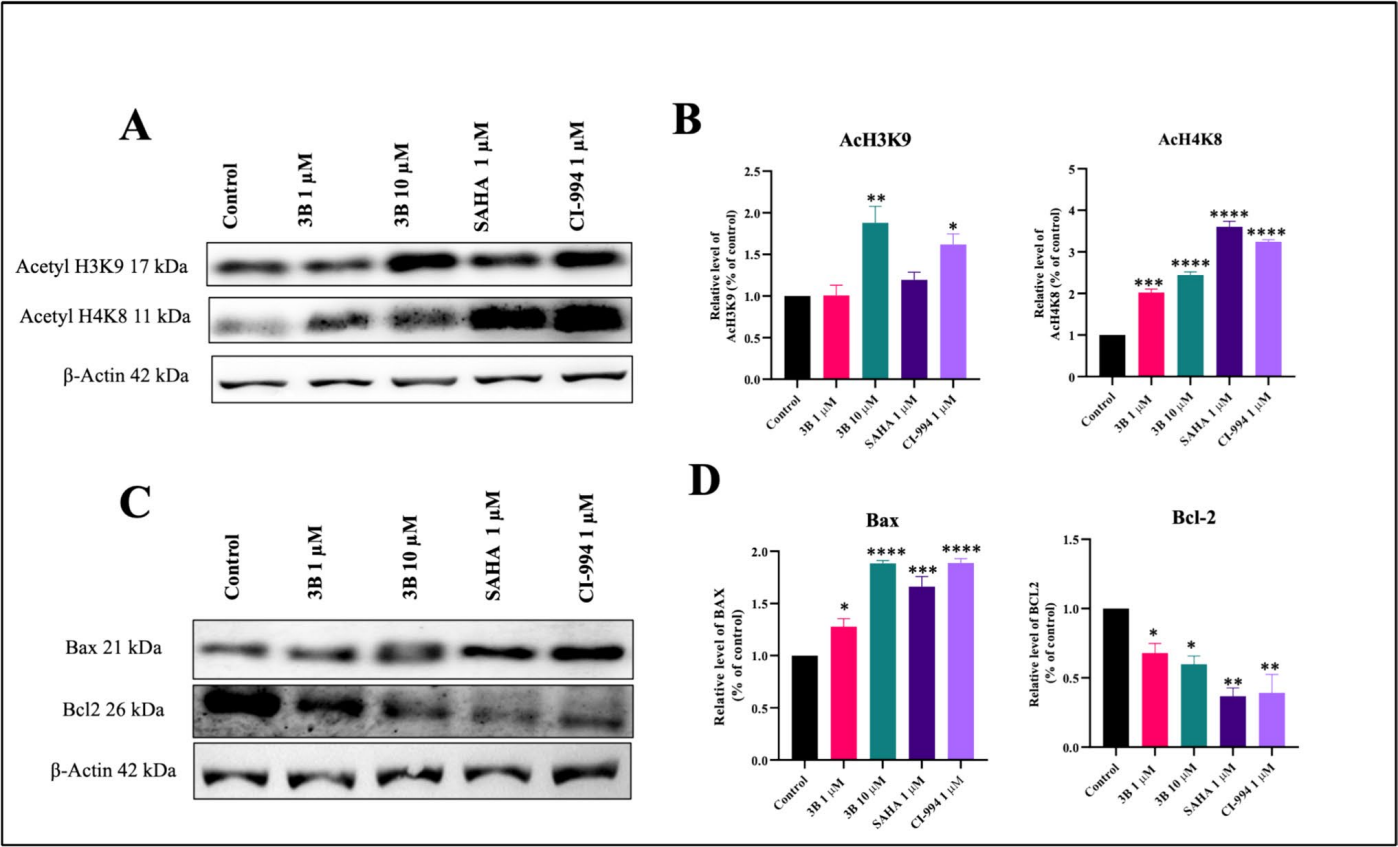
Western blot analysis to determine the effect of compounds on AcH3K9, AcH4K8, Bax, and Bcl-2 in SH-SY5Y cells treated with **3B**, SAHA, and CI-994 for 24 h. **A** Western blot analysis of effects of compounds on AcH3K9 and AcH4K8. **B** Densitometric quantification of H3K9 and H4K8 acetylation in cells treated with **3B**, SAHA, and CI-994 for 24 h compared with control. **C** Western blot analysis to determine the effects of compounds on Bax and Bcl-2. **D** Densitometric quantification of Bax and Bcl-2 in cells treated with **3B**, SAHA, and CI-994 for 24 h compared to the control, in cells treated with **3B**, SAHA, and CI-994 for 24 h compared to the control. *p < 0.05, **p < 0.01, ***p < 0.001, ****p < 0.0001 (*vs*. the control) determined with one-way ANOVA, Dunnett’s multiple comparison test

Considering the HDAC1 and HDAC2 activity and anticancer properties of compound 3B, the compound was selected for western blotting analysis and in vivo experiments. The hyperacetylation of histone H3 and H4 proteins was analyzed for 24 h. This study specifically examined the effects of compound **3B** on the acetylated histone markers AcH3K9 and AcH4K8. Additionally, the apoptotic pathway was analyzed by measuring the protein expression of pro-apoptotic protein Bax and anti-apoptotic protein Bcl-2, in both treated and untreated groups. The results demonstrated increase in AcH3K9 and AcH4K8 compared to control (Fig. 8). β-actin protein was used as a negative control and remained constant. Moreover, western blot analysis demonstrated a notable upregulation in the pro-apoptotic protein Bax, accompanied by a simultaneous downregulation of the anti-apoptotic protein Bcl-2 in the treated groups contrast to the control group (Fig. 8). These findings indicate that the compound **3B** induces apoptosis as HDACi with pro-apoptotic effects.

### In vivo antitumor activity

**Fig. 9:**
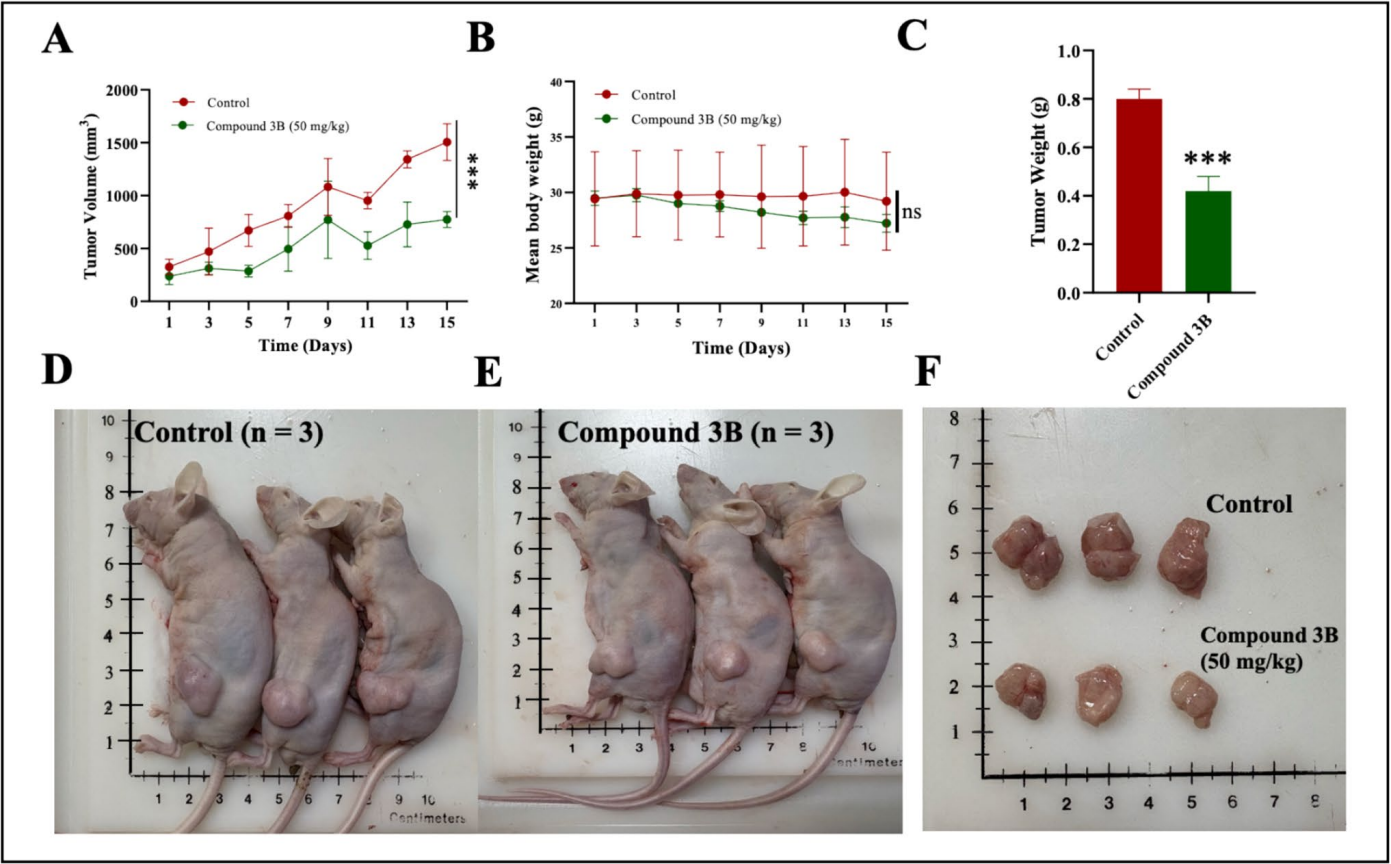
In vivo antitumor efficacy of compound **3B** against the SH-SY5Y xenograft model. **A** Tumor volume measurement between the control and treated group. **B** Body weight comparison between the control and treated groups. **C** Tumor weight comparison between control and treated group. **D** Images showing tumor-bearing nude mice in the untreated control group. **E** Images showing tumor-bearing nude mice treated with compound **3B** (50 mg/kg). **F** Excised tumors from both groups. Data are shown as the mean ± SD (*n* = 3). *p* values: ns: non-significant, ***p < 0.001. Determined with one-way ANOVA, Dunnett’s multiple comparison test

SH-SY5Y neuroblastoma xenograft model was developed in BALB/c nude male mice to assess the in vivo antitumor efficacy of compound **3B**. As shown in Fig. 9A, treatment with 50 mg/kg of compound **3B** significantly suppressed tumor development. Additionally, toxicity was assessed by monitoring changes in body weight. In this study, no significant changes were observed, indicating that compound **3B** was well tolerated at the administered dose (Fig. 9B). Furthermore, tumor weight was markedly lower than control group (Fig. 9C). The tumor growth inhibition (TGI) value of compound **3B** is 65% compared to the control. Figure 9D and E display images of tumor-bearing nude mice from both the untreated control and treated groups. A comparison of tumors between the control and treated groups is presented in Fig. 9F.

### Histopathological analysis

**Fig. 10:**
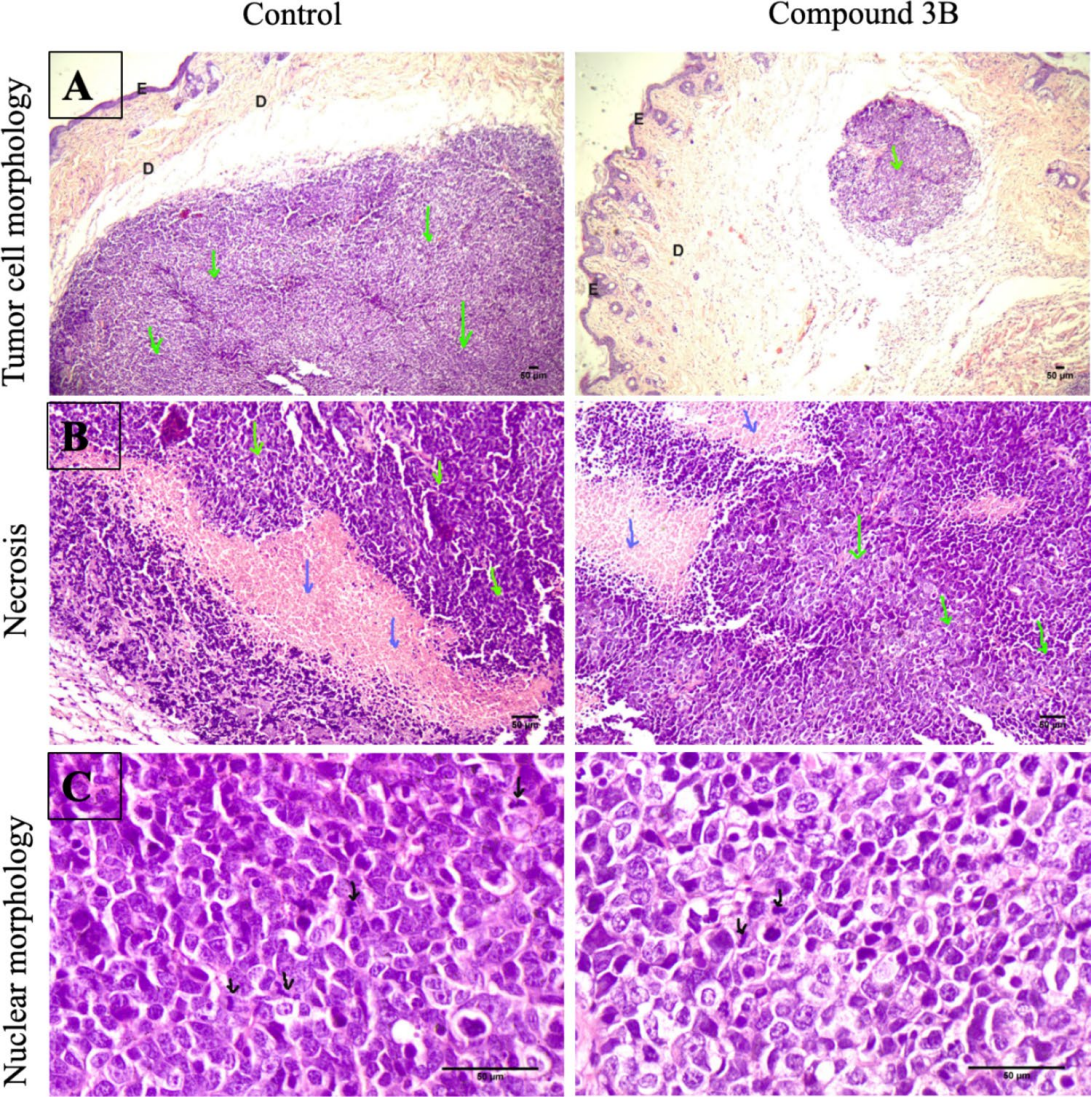
Histopathological assessment after compound **3B** treatment in in vivo model. Morphological analysis of tumor control and test compound **3B** via hematoxylin and eosin (H&E) staining. Representative images showing H&E-stained sections of the control and treatment group. **A** Tumor cell morphology represented by the green arrow (40 ×). **B** Tumor area represented by the green arrow and necrosis area represented by the blue arrow (100 ×). **C** Nuclear morphology with mitotic figure represented in black arrow (epidermis—E; dermis—D; 400 ×) (scale bars 50 μm)

Morphological parameters such as tumor cell morphology, necrosis, and mitotic figures in the tumor were analyzed. Tumor sections revealed a well-circumscribed nodular lesion occupying the papillary and reticular dermis, extending into the subcutaneous region. The tumor cells, which were arranged in a trabecular growth pattern, consist of small, round, blue cells with moderate cytoplasm and display nuclear atypia, including cellular and nuclear pleomorphism (variations in cell and nuclear size and shape), hyperchromatic nuclei (intensely stained due to excessive chromatin), an increased nuclear-to-cytoplasmic ratio, and a salt-and-pepper chromatin pattern. The nucleus and cytoplasm features are areas of necrosis, characterized by the loss of cellular and nuclear details. In contrast to the control group, the test group demonstrated a slight decreases in tumor infiltration and nuclear atypia. Furthermore, the test group revealed a slight reduction in mitotic compared with that of the control group (Fig. 10A–C).

## Discussion

Over the past decades, neuroblastoma has remained one of the most aggressive pediatric cancers, often classified as high-risk and frequently exhibiting resistance to conventional therapies. These challenges have driven research toward the discovery of novel small-molecule inhibitors and alternative treatment strategies [16]. Among the most promising approaches are epigenetic interventions that target DNA methyltransferases, histone acetyltransferases, and HDACs. In this context, HDACis have emerged as a promising class of epigenetic drugs capable of overcoming therapeutic resistance in neuroblastoma by reactivating silenced tumor suppressor genes and promoting apoptosis [17].

Among the HDAC family, HDAC1 and HDAC2 are among the most extensively studied isoforms due to their important roles in embryonic development, neurological function, and cancer progression. These enzymes are required for neural cell proliferation and differentiation; however, their overexpression has been linked not only to tumorigenesis but also neurological disorders such as Parkinsons and Alzheimer’s [18]. HDAC1 is mainly expressed in progenitor cells, non-neuronal support cells, and certain mature neurons, whereas HDAC2 is predominantly found in post-mitotic immature neurons and is downregulated in mature neurons [19]. Understanding of the spatial and temporal expression patterns of HDAC1 and HDAC2 is crucial for the development of targeted therapies. Several research works have demonstrated that their overexpression causes enhanced proliferation in neuroblastoma cells, indicating the urgent need for selective HDAC1/2 inhibitors as potential therapeutic agents to reduce tumor growth while minimizing neurological side effects.

In recent years, HDAC-targeted drugs have been focused on improving their efficacy and reducing side effects [20]. Structural optimizations, driven by both computational modelling and conventional approaches, have led to improved drug design [21]. SAHA, a well-known hydroxamic acid-based HDACi, has been widely used to study its mechanism of action across various cell lines. Previous studies have shown that modifications to the linker and cap group to enhance drug selectivity and binding affinity. In current study, the synthesized compounds also have hydroxamic acid moiety, with variations introduced in the cap group. This study focused on two novel hydroxamic acid-based analogues, compounds **3A** and **3B**, which demonstrated potent anticancer activity against SH-SY5Y neuroblastoma cells. Cell cycle analysis revealed that compound **3B** induced significant G2/M phase arrest, effectively inhibiting cell growth and proliferation. The colony formation assay confirmed reduction in colony efficiency followed by treatment with compounds **3A** and **3B**, indicating their anti-proliferative potential. Confocal imaging revealed nuclear bulging, a hallmark of apoptosis, further confirming the compounds’ effect on cellular architecture and programmed cell death. Another similar study shown that HDACis exert their therapeutic effects by promoting histone acetylation, thereby increasing the transcription of genes involved in cell cycle regulation, including those controlling the G1 and G2/M phases [22]. Compounds **3A** and **3B** resulted in potent inhibition of HDAC1 and HDAC2, suggesting their potential to effectively reduce the neuroblastoma growth. The results of the western blotting analysis confirmed compound **3B** effectively increased histone acetylation (AcH3K9 and AcH4K8) while modulating apoptosis-related proteins by upregulating Bax and downregulating Bcl-2, validating its HDAC-inhibitory and pro-apoptotic effects. Similar studies in B cell lymphomas, with HDACis TSA, and sodium butyrate have also been shown to induce apoptosis by downregulating Bcl-2 [23]. Panicker and team [24] also demonstrated that HDACis directly trigger apoptosis in neuroblastoma cells, as evidenced by PARP cleavage following treatment with HDACis like TSA and Romidepsin. Apoptosis induction has been linked to the inverse regulation of pro-apoptotic (Bax) and anti-apoptotic (Bcl-x) proteins, suggesting a mechanism by which HDACis enhance cell death [25].

Since the compounds were potent in in vitro, we focused on the in vivo study, where xenograft experiments showed that compound **3B** revealed a significant reduction in tumor size without affecting body weight. Histopathological analysis further confirmed its potent antitumor activity, showing reduced tumor volume and increased necrosis in the treatment group. In a similar study, compound 14 d, synthesized by Yun et al. [26] as a dual HDAC1/2 and CDK2 inhibitor, exhibited a 40% TGI at 50 mg/kg, showing superior efficacy compared with control group [26]. In comparison, the present in vivo study showed that compound **3B** exhibited a 65% TGI at 50 mg/kg indicating potent anticancer property. Recent studies have demonstrated that HDAC1 and HDAC2 play a critical role in the aggressiveness of neuroblastoma; however, the development of HDACis that suppress neuroblastoma proliferation is less explored.

## Conclusion

In summary, compound **3B** is a potent and promising HDAC1 and HDAC2 inhibitor, with potent anticancer activity against neuroblastoma. Given its strong in vitro and in vivo efficacy, it represents a potential therapeutic candidate for further development in neuroblastoma treatment.

## Data Availability

No datasets were generated or analysed during the current study.
